# Perioperative Blood Transfusion as a Poor Prognostic Factor After Aggressive Surgical Resection for Hilar Cholangiocarcinoma

**DOI:** 10.1007/s11605-014-2741-8

**Published:** 2015-01-21

**Authors:** Norihisa Kimura, Yoshikazu Toyoki, Keinosuke Ishido, Daisuke Kudo, Yuta Yakoshi, Shinji Tsutsumi, Takuya Miura, Taiichi Wakiya, Kenichi Hakamada

**Affiliations:** Department of Gastroenterological Surgery, Hirosaki University Graduate School of Medicine, 5 Zaifu-cho, Hirosaki City, Aomori Province 036-8562 Japan

**Keywords:** Hilar cholangiocarcinoma, Aggressive surgery, Hepatectomy, Prognosis, Blood transfusion

## Abstract

**Background:**

Blood transfusion is linked to a negative outcome for malignant tumors. The aim of this study was to evaluate aggressive surgical resection for hilar cholangiocarcinoma (HCCA) and assess the impact of perioperative blood transfusion on long-term survival.

**Methods:**

Sixty-six consecutive major hepatectomies with en bloc resection of the caudate lobe and extrahepatic bile duct for HCCA were performed using macroscopically curative resection at our institute from 2002 to 2012. Clinicopathologic factors for recurrence and survival were retrospectively assessed.

**Results:**

Overall survival rates at 1, 3, and 5 years were 86.7, 47.3, and 35.7 %, respectively. In univariate analysis, perioperative blood transfusion and a histological positive margin were two of several variables found to be significant prognostic factors for recurrence or survival (*P* < 0.05). In multivariate analysis, only perioperative blood transfusion was independently associated with recurrence (hazard ratio (HR) = 2.839 (95 % confidence interval (CI), 1.370–5.884), *P* = 0.005), while perioperative blood transfusion (HR = 3.383 (95 % CI, 1.499–7.637), *P* = 0.003) and R1 resection (HR = 3.125 (95 % CI, 1.025–9.530), *P* = 0.045) were independent risk factors for poor survival.

**Conclusions:**

Perioperative blood transfusion is a strong predictor of poor survival after radical hepatectomy for HCCA. We suggest that circumvention of perioperative blood transfusion can play an important role in long-term survival for patients with HCCA.

## Introduction

Surgical resection^1^,^2^ or liver transplantation^3^ remains the only curative treatment that can offer long-term survival for hilar cholangiocarcinoma (HCCA). HCCA involves the confluence of the bilateral hepatic ducts where the main portal and hepatic arterial branches are close, and thus major hepatectomy is often required to obtain a disease-free margin status. Simultaneous caudate lobectomy with extended resection of the hilar bile ducts is now the procedure of choice because the bile ducts of the caudate lobe are direct tributaries from the bilateral hepatic ducts.^4^,^5^ In fact, several reports have shown that hepatectomy with en bloc resection of the caudate lobe and extrahepatic bile duct,^6^,^7^ lymph node dissection,^8^ or resection of portal vein^9^,^10^ or hepatic artery^11^,^12^ for HCCA is more likely to result in disease-free resection. Preoperative percutaneous transhepatic portal vein embolization (PTPVE) has also helped to make major hepatectomy safer and applicable to more patients.^13^^–^ 18

Despite these preoperative and surgical advances for achieving disease-free resection, many patients still have microscopic positive resection margins. This is of concern because margin status is a critical prognostic factor and cases with microscopic invasive carcinoma at the ductal margin have poor survival.^6^,^7^,^16^,^17^,^19^ However, aggressive and complex procedures such as hepatectomy with en bloc resection of the caudate lobe and extrahepatic bile duct for HCCA cause excessive intraoperative loss of blood that requires perioperative blood transfusion, which may also result in negative outcomes for malignant tumors of the liver.^20^ However, the effects of blood transfusion on survival after aggressive surgery for HCCA have not been widely studied.

In this study, we surveyed retrospective data in 66 consecutive hepatectomies with en bloc resection of the caudate lobe and extrahepatic bile duct for HCCA performed at our institute. The main aim was to identify prognostic factors for recurrence and long-term survival after aggressive surgery for HCCA, with a particular focus on perioperative blood transfusion.

## Materials and Methods

### Patients

Between January 2002 and December 2012, 108 patients with HCCA were admitted to the Department of Gastroenterological Surgery at Hirosaki University Hospital. Nineteen patients did not undergo any surgery due to highly advanced unresectable disease or poor liver function during preoperative workup. Laparotomy was conducted in the remaining 89 patients. Twenty-three of the patients were excluded because three patients underwent minor hepatic resection (only segment 4) with caudate lobe and extrahepatic bile duct; one patient had R2 (macroscopic residual tumor) despite left hepatectomy with caudate lobe and extrahepatic bile duct; nine patients underwent palliative bile duct resection; two patients underwent palliative pancreatoduodenectomy; and eight patients were not able to undergo resection due to liver metastases or intraoperative detection of highly local advanced disease. The remaining 66 patents underwent major hepatectomies (hemihepatectomy, central bisectionectomy, or more extensive resection) with en bloc resection of the caudate lobe and extrahepatic bile duct in addition to macroscopically curative resection (R0 or R1 resection) were enrolled in this study. HCCA was defined as a tumor involving the primary ductal confluence. Tumors invading the hepatic hilar region but located predominantly in the liver parenchyma and gall bladder were not included in the definition of HCCA. All clinical information was collected based on prospectively maintained data and analyzed retrospectively. Demographic data included information on gender, age, mode of presentation, pre-resection interventions, and investigations. Preoperative laboratory values included serum carbohydrate antigen 19-9 (CA19-9) as a tumor marker and indocyanine green retention value at 15 min (indocyanine green (ICG)-R15) as a marker of hepatic function reserve. Operative records were reviewed for operative details, operative time, intraoperative estimated blood loss, and blood transfusion requirements.

### Preoperative Evaluation and Workup

The location and extent of the disease were evaluated by ultrasonography, enhanced computed tomography (CT), magnetic resonance imaging (MRI) with magnetic resonance cholangiopancreatography (MRCP), endoscopic retrograde cholangiography (ERC), and percutaneous transhepatic cholangiography (PTC). Enhanced CT was performed before biliary drainage, which in turn was performed if obstructive jaundice (total bilirubin (T-bil) >3 mg/dL) was present. Endoscopic biliary drainage (EBD) was preferred, but percutaneous transhepatic biliary drainage (PTBD) was performed when EBD was not successful. Biliary drainage was performed primarily for the future liver remnant (FLR) lobe; however, in cases of bilateral biliary sepsis, both lobes were drained. Biliary drainage was undertaken until T-bil decreased to <2 mg/dL. In patients with cholangitis, surgery was postponed until alleviation of inflammation. Enhanced CT of the chest, abdomen, and pelvis was used to identify metastatic lesions. More recently, positron emission tomography (PET) has also been used for this purpose. Unresectable disease was defined as the presence of extensive bilobular metastases or extrahepatic metastases other than regional lymph node involvement or peritoneal dissemination.

The total liver volume (TLV) and the part of the hepatic segment to be resected were calculated using CT volumetry.^21^ PTPVE was indicated when the FLR was estimated to be <40 % and was performed 3 to 4 weeks before scheduled liver resection, since PTPVE has been suggested to be useful for inducing compensatory hypertrophy of the FLR.^13^ All patients underwent CT within 4 weeks after PTPVE. ICG tests were performed in all cases before surgery. For patients who underwent PTPVE, ICG tests were performed just before and 3 to 4 weeks after PTPVE. ICG (0.5 mg/kg body weight) was administered via a peripheral vein and venous blood was sampled before and 5, 10, and 15 min after injection. The plasma clearance rate of ICG (KICG) was calculated by linear regression analysis of plasma ICG concentrations.^22^ Total liver function was evaluated based on KICG, and the KICG of the FLR was calculated using the following formula: KICG × FLR volume/TLV.^18^

### Surgical Technique

All operations were performed after T-bil had decreased to <2 mg/dL. Preoperative autologous blood donation was performed in patients with preoperative hemoglobin level of >11 g/dL and no hypotension or severe cardiac disease, at the discretion of the anesthesiologist. Firstly, en bloc dissection of the regional lymph nodes surrounding the hepatoduodenal ligament, behind the pancreatic head, and around the common hepatic artery was uniformly performed, followed by skeletonization of the hepatic hilus. After complete mobilization of the hepatic lobe to be resected, the caudate lobe was completely separated from the inferior vena cava. Liver transection was carried out using CUSA (Aloka, Tokyo, Japan) or the forceps clamp-crushing method based on the surgeon’s preference during hepatic artery and portal vein clamping for 15 min with 5-min intervals, which is known as Pringle’s maneuver. All patients underwent hemihepatectomy, central bisectionectomy, or more extensive resection with en bloc resection of the caudate lobe and extrahepatic bile duct. Biliary continuity was reconstructed with hepaticojejunostomy using a Roux-en-Y jejunal limb brought up in a transmesenteric fashion. Combined pancreatoduodenectomy was performed if the tumor extended below the intrapancreatic portion of the distal bile duct.^23^ Reconstruction for hepatopancreatoduodenectomy was conducted by a modified Child’s method with an end-to-side anastomosis. Concomitant portal vein resection and reconstruction have been applied aggressively in cases with suspected macroscopic invasion during surgery.^9^,^10^ Concomitant hepatic artery resection and reconstruction have been similarly applied, although this is still controversial.^11^,^12^,^24^^–^ 26 All autologous blood collected preoperatively was transfused intraoperatively. Allogeneic red blood cells and fresh frozen plasma (FFP) were transfused intraoperatively at the discretion of the anesthesiologist.

### Histopathological Evaluation

Pathology reports were reviewed to determine tumor histological grade, margin status, and the presence of microvascular, lymphovascular and perineural invasion. Tumors were staged using the tumor-node-metastasis (TNM) Classification of Malignant Tumors of the International Union Against Cancer (7th edition, 2009) for proximal extrahepatic bile duct cancer.^27^ Therefore, intrahepatic metastasis, peritoneal deposits and positive para-aortic nodes were classified as distant metastasis (M1). R0 resection was defined as negative ductal and radial margins on histology; R1 resection as the histological presence of a tumor at any margin; and R2 resection as the macroscopic presence of a tumor at any margin or M1. Histopathological evidence of invasive carcinoma, dysplastic lesions including carcinoma in situ (CIS), or normal epithelium was recorded for proximal and distal ductal margins. Radial margins were assessed in the resected specimen only. Dysplastic lesions and CIS were defined as R0 resection because it is difficult to distinguish CIS from high-grade dysplastic lesions pathologically,^28^ and the presence of CIS at the ductal margin has no impact on survival compared with a negative ductal margin in extrahepatic cholangiocarcinoma.^29^^–^ 34

### Postoperative Course

Patients were not routinely admitted to the intensive care unit postoperatively but were monitored overnight in the general ward. In principle, allogeneic red blood cell transfusion was performed for asymptomatic patients if the hemoglobin level fell to <6.5 g/dL. However, patients with additional specific risk factors such as severe cardiac disease or hypotension often received blood transfusion when the hemoglobin level fell to <7 g/dL. FFP was used at the discretion of the attending surgeon and was most commonly administered for prothrombin time (PT) <60 %. Liver function tests were obtained routinely on postoperative days 1, 3, and 6 and as clinically indicated thereafter. Hepatic failure was defined as T-bil of >7.0 mg/dL postoperatively, and hyperbilirubinemia was defined as T-bil of >5.0 mg/dL.^35^ Hence, the postoperative maximum T-bil and minimum PT were assessed. Postoperative complications were defined and graded according to the validated Clavien classification system^36^ as grades 1 and 2 and grades 3–5 for minor and major complications, respectively. Postoperative mortality was defined as death as an inpatient or within 30 days of surgery.

### Adjuvant Treatment

Since 2008, all patients except UICC stage I cases were scheduled to receive S-1, an oral fluoropyrimidine derivative, as adjuvant chemotherapy for about 1 year following surgery, whereas those prior to 2007 did not receive chemotherapy.

### Follow-up After Surgical Resection

Patients were followed regularly at the outpatient clinic every 1–3 months. Clinical examinations were performed, tumor markers (carcinoembryonic antigen and CA19-9) and liver function were checked monthly, and CT scans of the chest, abdomen, and pelvis were performed every 3 months until year 2. Thereafter, clinical examinations, tumor marker tests, liver function tests, and CT scans were carried out every 6 months until year 5. Subsequently, patients attended a clinic for an annual examination. MRI and PET-CT were carried out if recurrence was suspected in routine follow-up.

### Survival and Prognostic Factors

The following data were reviewed to identify prognostic factors: (1) preoperative clinical factors: gender, age, jaundice, preoperative CA19-9 value, preoperative ICG-R15, FLR ratio, KICG of the FLR, and preoperative PTPVE; (2) treatment-related factors: operative procedure, operative time, operative blood loss, perioperative blood transfusion (except for autologous blood transfusion), postoperative complication, postoperative maximum T-bil, postoperative minimum PT, and adjuvant chemotherapy; and (3) pathologic factors: T-stage (UICC), lymph node metastasis, tumor histological grade, microvascular invasion, lymphovascular invasion, perineural invasion, and resection margin status.

Disease-free survival (DFS) was defined as the time from the operation to the date of disease recurrence. Overall survival (OS) was defined as the time from the operation to the time of death due to recurrence or the last follow-up time.

### Statistical Analysis

For variables associated with perioperative blood transfusion, continuous variables are expressed as median and interquartile range and were compared by Mann–Whitney *U* test, and categorical variables were compared by Pearson Chi-square test with a Fisher exact test or Yates continuity correction where appropriate in univariate analysis. Variables with a significant relationship with perioperative blood transfusion in univariate analysis were used in a multivariate logistic regression model. DFS and OS were calculated by the Kaplan–Meier method, and differences were evaluated by log-rank test. Only variables that were significant in univariate analysis were included in multivariate analysis to identify independent predictors of DFS and OS. This analysis was performed using a Cox proportional hazards regression model. *P* < 0.05 was considered significant in all analyses. All analyses were performed using PASW Statistics ver.18.0 for Windows (SPSS, Inc., Chicago, IL, USA).

## Results

### Patient Characteristics and Preoperative Management

The characteristics of the 66 patients (43 males, 23 females) are summarized in Table 1. The patients had a median age of 68.5 years (range, 39–80 years), and all underwent major hepatectomy with en bloc resection of the caudate lobe and extrahepatic bile duct for HCCA. Jaundice at initial presentation was present in 47 patients (71.2 %). Biliary drainage was performed in 60 patients (90.9 %), including 13 nonjaundiced patients who underwent biliary drainage to relieve cholangitis or to determine the extent of a lesion along the individual intrahepatic segmental duct. EBD, PTBD, and both EBD and PTBD were performed in 32 (53.3 %), 24 (40.0 %), and 4 cases (6.7 %), respectively. The median CA 19-9 value was 38.5 U/mL (range, 3–14,567 U/mL), and the median ICG-R15 was 10.8 % (0.1–43 %). The median FLR ratio and KICG of FLR were 47.5 % and 0.070, respectively. PTPVE was carried out in 32 patients (48.5 %).

**Table 1:** Patient characteristics and outcomes

	Total (*n* = 66)	No transfusion (*n* = 37)	Transfusion (*n* = 29)	*P* value
Gender				
Male	43 (65.2 %)	21 (72.4 %)	22 (59.5 %)	0.273^b^
Female	23 (34.8 %)	8 (27.6 %)	15 (40.5 %)	
Age	68.5 (39–80)	66 (39–79)	70 (42–80)	0.096^a^
Preoperative jaundice				
No	19 (28.8 %)	14 (37.8 %)	5 (17.2 %)	0.067^b^
Yes	47 (71.2 %)	23 (62.2 %)	24 (82.8 %)	
Preoperative serum value				
CA19-9 (U/mL)	38.5 (3–14,567)	20 (3–14,567)	62 (3–6666)	0.039^a,^ *
ICG-R15 ( %)	10.75 (0.1–43)	10.8 (0.7–43.0)	9.0 (0.1–32.9)	0.574^a^
FLR ratio ( %)	47.5 (25.1–86.3)	46.5 (25.1–86.3)	50.4 (33.4–77.0)	0.179^a^
KICG of FLR	0.070 (0.021–0.323)	0.069 (0.021–0.179)	0.073 (0.028–0.323)	0.275^a^
Preoperative PTPVE				
No	34 (51.5 %)	21 (56.8 %)	13 (44.8 %)	0.336^b^
Yes	32 (48.5 %)	16 (43.2 %)	16 (55.2 %)	
Operative details				
Right hepatectomy	43 (65.2 %)	24 (64.9 %)	19 (65.5 %)	0.337^b^
Left hepatectomy	19 (28.8 %)	9 (24.3 %)	10 (34.5 %)	
Right trisectionectomy	3 (4.5 %)	3 (8.1 %)	0 (0.0 %)	
Central bisectionectomy	1 (1.5 %)	1 (2.7 %)	0 (0.0 %)	
Operative time (min)	500.5 (307–710)	470 (307–670)	590 (390–710)	<0.001^a,^ *
Operative blood loss (mL)	1778.5 (250–11,170)	1380 (250–2800)	2910 (990–11,170)	<0.001^a,^ *
Postoperative complication (Clavien–Dindo classification)				
Grade 0	4 (6.1 %)	4 (10.8 %)	0 (0.0 %)	0.197^b^
Grade 1	9 (13.6 %)	7 (18.9 %)	2 (6.9 %)	
Grade 2	22 (33.3 %)	10 (27.0 %)	12 (41.4 %)	
Grade 3	26 (39.4 %)	14 (37.8 %)	12 (41.4 %)	
Grade 4	4 (6.1 %)	2 (5.4 %)	2 (6.9 %)	
Grade 5 (Hospital death)	1 (1.5 %)	0 (0.0 %)	1 (3.4 %)	
Postoperative max. T-bil (mg/dL)	3.55 (1.4–14.7)	2.8 (1.4–10.4)	4.8 (2.0–14.7)	<0.001^a,^ *
Postoperative min. PT ( %)	66.5 (34–93)	68 (38–93)	62 (34–89)	0.130^a^
Adjuvant chemotherapy				
Yes	30 (45.5 %)	20 (54.1 %)	10 (34.5 %)	0.113^b^
No	36 (54.5 %)	17 (45.9 %)	19 (65.5 %)	
T-stage (UICC)				
T1	4 (6.1 %)	3 (8.1 %)	1 (3.4 %)	0.542^b^
T2a	22 (33.3 %)	14 (37.8 %)	8 (27.6 %)	
T2b	21 (31.8 %)	11 (29.7 %)	10 (34.5 %)	
T3	12 (18.2 %)	7 (18.9 %)	5 (17.2 %)	
T4	7 (10.6 %)	2 (5.4 %)	5 (17.2 %)	
Lymph node status				
Negative	44 (66.7 %)	27 (73.0 %)	17 (58.6 %)	0.220^b^
Positive	22 (33.3 %)	10 (27.0 %)	12 (41.4 %)	
Tumor histological grade				
Papillary/well differentiated	16 (24.2 %)	8 (21.6 %)	8 (27.6 %)	0.575^b^
Moderate/poorly differentiated	50 (75.8 %)	29 (78.4 %)	21 (72.4 %)	
Microvascular invasion				
Negative	18 (27.3 %)	11 (29.7 %)	7 (24.1 %)	0.613^b^
Positive	48 (72.7 %)	26 (70.3 %)	22 (75.9 %)	
Lymphovascular invasion				
Negative	10 (15.2 %)	8 (21.6 %)	2 (6.9 %)	0.093^b^
Positive	56 (84.8 %)	29 (78.4 %)	27 (93.1 %)	
Perineural invasion				
Negative	10 (15.2 %)	7 (18.9 %)	3 (10.3 %)	0.271^b^
Positive	56 (84.8 %)	30 (81.1 %)	26 (89.7 %)	
Margin status				
R0	54 (81.8 %)	32 (86.5 %)	22 (75.9 %)	0.267^b^
R1	12 (18.2 %)	5 (13.5 %)	7 (24.1 %)	

Values are presented as *n* (%) or median (range)

*ICG-R15* indocyanine green retention rate at 15 min, *FLR* future liver remnant, *KICG* indocyanine green clearance rate, *PTPVE* percutaneous transhepatic portal vein embolization, *T-bil* total bilirubin, *PT* prothrombin time, *UICC* Union for International Cancer Control

^a^Mann–Whitney *U* test

^b^Chi-square test

*P < 0.05

### Treatment-Related Characteristics

The operative procedures included right hepatectomy (*n* = 43), left hepatectomy (*n* = 19), right trisectionectomy (*n* = 3), and central bisectionectomy (*n* = 1) with en bloc resection of the caudate lobe and extrahepatic bile duct. No patients underwent left trisectionectomy. Portal vein resection and hepatic artery resection were required in 11 (16.7 %) and 1 case (1.5 %), respectively, and seven patients (10.6 %) underwent combined pancreatoduodenectomy. The median operative time was 500.5 min (range, 307–710 min), and the median estimated blood loss was 1778.5 mL (range, 250–11,170 mL). Perioperative allogeneic red blood cell transfusion was performed in 29 patients (43.9 %). Postoperative major complications occurred in 31 patients (47 %), with intra-abdominal abscess requiring percutaneous drainage being the most frequent complication, occurring in 19 patients. Bile leakage from the liver transection surface, severe ascites, hepaticojejunostomy insufficiency, and pleural effusion requiring an invasive intervention occurred in 11, 10, 9, and 8 patients, respectively. In two patients, radiologic intervention was performed due to postoperative bleeding from pseudoaneurysm of the hepatic artery. The mortality rate was 1.5 % (one patient), and this patient died of sepsis related to intra-abdominal abscess with breakdown of hepaticojejunostomy. The postoperative maximum T-bil was 3.6 mg/dL (range, 1.4–14.7 mg/dL), with 12 patients diagnosed with hepatic failure and 19 with hyperbilirubinemia. The postoperative minimum PT was 66.5 % (range, 34–93 %). Adjuvant chemotherapy was given postoperatively in 30 patients (45.5 %), using S-1 and gemcitabine in 28 and 2 patients, respectively.

### Tumor Characteristics

Four patients (6.1 %) had stage T1 disease, 22 (33.3 %) had T2a, 21 (31.8 %) had T2b, 12 (18.2 %) had T3, and 7 (10.6 %) had T4. Lymph node infiltration was noted in 22 patients (33.3 %). For the histological tumor grade, papillary or well-differentiated adenocarcinomas were found in 16 patients (24.2 %) and moderate or poorly differentiated tumors were present in 50 (75.8 %). Microvascular, lymphovascular, and perineural invasion were seen in 48 (72.7 %), 56 (84.5 %), and 56 (84.8 %) patients, respectively. R0 resection was achieved in 54 patients (81.8 %) and R1 resection in 12 (18.2 %).

### Risk Factors for Perioperative Blood Transfusion

Of the 66 patients, 29 (43.9 %) and 37 (56.1 %) did and did not receive perioperative allogeneic red blood cell transfusion, respectively. A median of 4 blood units were transfused (range, 1–32 units). The clinical, operative, and pathological differences between these two groups of patients are shown in Table 1. Blood transfusion was more frequent in patients with a high preoperative CA19-9 level (median, 62 vs. 20 U/mL, *P* = 0.039). Patients who received blood transfusion also had longer operation times (median, 590 vs. 470 min, *P* < 0.001), higher estimated blood loss (median, 2910 vs. 1380 mL, *P* < 0.001), and higher postoperative maximum T-bil (median, 4.8 vs. 2.8 mg/dL, *P* < 0.001). In multivariate analysis, only the estimated blood loss was independently associated with blood transfusion (odds ratio = 1.002 (95 % confidence interval (CI), 1.001–1.003), *P* = 0.002) (Table 2).

**Table 2:** Significant factor for perioperative blood transfusion in multivariate analysis

Variable	Odds ratio	95 % CI	*P* value
Preoperative CA19-9 value (U/mL)	1.000	0.999–1.000	0.337
Operative time (min)	1.002	0.994–1.011	0.583
Operative blood loss (mL)	1.002	1.001–1.003	0.002*
Postoperative maximum T-bil (mg/dL)	1.205	0.914–1.589	0.186

*95% CI* 95 % confidence interval

*P < 0.05

### Survival Analysis

The median follow-up was 23.0 months (range, 1.2–109.0 months). OS rates at 1, 3, and 5 years for the entire cohort were 86.7, 47.3, and 35.7 %, respectively, with a median OS of 31.7 months. The 1-, 3-, and 5-year DFS rates for the same population were 66.3, 40.5, and 30.6 %, respectively, with a median DFS of 20.3 months.

**Fig. 1:**
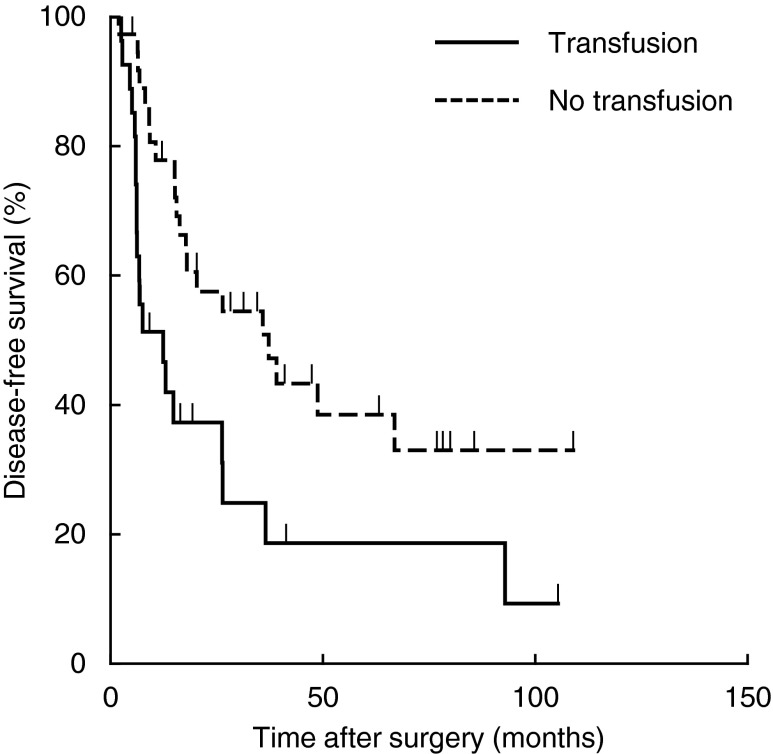
Disease-free survival curves for patients after aggressive surgical resection for hilar cholangiocarcinoma stratified by perioperative allogeneic red blood cell transfusion status. Median time to recurrence of 37.2 months without transfusion vs. 12.3 months with transfusion, *P* = 0.007

The clinical, operative and pathological factors influencing DFS are shown in Table 3. In univariate analysis, significant predictors of decreased DFS were male gender (*P* = 0.027), preoperative CA19-9 >37 U/mL (*P* = 0.004), preoperative PTPVE (*P* = 0.029), operative procedures except for left hepatectomy (*P* = 0.038), perioperative blood transfusion (*P* = 0.007), postoperative maximum T-bil >5.0 mg/dL (*P* = 0.026), positive lymph node (*P* = 0.001), microvascular invasion (*P* = 0.005), and lymphovascular invasion (*P* = 0.048). In multivariate analysis, perioperative blood transfusion was the only independent prognostic factor for DFS (hazard ratio (HR) = 2.839, 95 % CI 1.370–5.884, *P* = 0.005). Kaplan–Meier survival curves gave median times to recurrence after resection of 12.3 and 37.2 months with and without blood transfusion, respectively (Fig. 1).

**Table 3:** Clinicopathological features predicting disease-free survival

Variable	Number	3-year DFS (%)	MST (month)	Univariate analysis	Multivariate analysis		
				*P* value	RR	95 % CI	*P* value
Gender							
Male	43	32.9	15.1	0.027*	2.007	0.849–4.748	0.113
Female	23	54.9	36.5				
Age							
≤70 years	42	41.9	17.9	0.543			
>70 years	24	34.4	20.3				
Preoperative jaundice							
No	19	50.0	26.4	0.118			
Yes	47	35.7	16.3				
Preoperative serum CA19-9							
≤37 U/mL	32	61.7	39.1	0.004*	1.903	0.834–4.339	0.126
>37 U/mL	34	17.9	12.3				
Preoperative ICG-R15							
≤15 %	48	49.0	26.4	0.063			
>15 %	18	17.4	15.1				
FLR ratio							
>40 %	50	39.3	20.3	0.976			
≤40 %	16	43.8	17.9				
KICG of FLR							
>0.05	53	42.5	20.2	0.356			
≤0.05	13	31.7	17.7				
Preoperative PTPVE							
No	34	51.9	36.5	0.029*	0.899	0.376–2.150	0.811
Yes	32	27.4	12.9				
Liver resection							
Left	19	61.1	48.8	0.038*	1.518	0.527–4.368	0.439
Right^a^	47	30.6	15.6				
Operative time							
≤480 min	28	37.8	20.3	0.819			
>480 min	38	42.3	16.3				
Operative blood loss							
≤1500 mL	26	47.7	35.8	0.245			
>1500 mL	40	37.3	14.8				
Perioperative blood transfusion							
No	37	50.9	37.2	0.007*	2.839	1.370–5.884	0.005*
Yes	29	24.9	12.3				
Postoperative complication (Clavien–Dindo classification)							
Grades 0–2	35	47.0	35.8	0.055			
Grades 3–5	31	32.1	12.3				
Postoperative max. T-bil							
≤5.0 mg/dL	47	50.7	36.5	0.026*	0.655	0.292–1.467	0.304
>5.0 mg/dL	19	9.4	15.1				
Postoperative min. PT							
>60 %	46	37.9	20.3	0.964			
≤60 %	20	49.0	17.9				
Adjuvant chemotherapy							
Yes	30	37.8	20.3	0.802			
No	36	41.3	26.3				
T-stage (UICC)							
T1, T2	47	45.3	26.3	0.116			
T3, T4	19	24.1	15.1				
Lymph node status							
Negative	44	49.7	35.8	0.001*	1.832	0.796–4.213	0.154
Positive	22	21.9	6.8				
Tumor histological grade							
Papillary/well differentiated	16	35.0	20.3	0.630			
Moderate/poorly differentiated	50	41.6	17.7				
Microvascular invasion							
Negative	18	62.5	92.9	0.005*	2.620	0.841–8.166	0.097
Positive	48	31.6	15.1				
Lymphovascular invasion							
Negative	10	60.0	NR	0.048*	0.608	0.159–2.328	0.468
Positive	56	36.3	16.3				
Perineural invasion							
Negative	10	55.6	92.9	0.148			
Positive	56	38.0	15.6				
Margin status							
R0	54	43.5	26.4	0.091			
R1	12	24.9	12.9				

*DFS* disease-free survival, *MST* median survival time, *RR* risk ratio, *95 % CI* 95 % confidence interval, *ICG-R15* indocyanine green retention rate at 15 min, *FLR* future liver remnant, *KICG* indocyanine green clearance rate, *PTPVE* percutaneous transhepatic portal vein embolization, *T-bil* total bilirubin, *PT* prothrombin time, *UICC* Union for International Cancer Control

*P < 0.05

^a^Including three right trisectionectomy and one central bisectionectomy

**Fig. 2:**
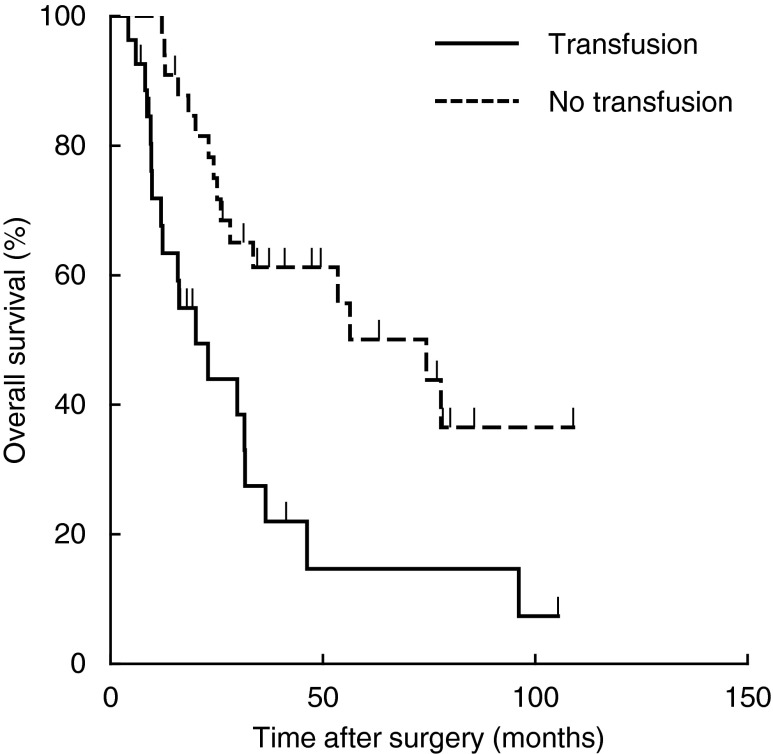
Overall survival curves for patients after aggressive surgical resection for hilar cholangiocarcinoma stratified by perioperative allogeneic red blood cell transfusion status. Median survival time of 74.3 months without transfusion vs. 20.1 months with transfusion, *P* = 0.002

The clinical, operative and pathological factors influencing OS are shown in Table 4. In univariate analysis, significant predictors of decreased OS were male gender (*P* = 0.033), preoperative jaundice (*P* = 0.010), preoperative CA19-9 >37 U/mL (*P* = 0.004), preoperative PTPVE (*P* = 0.035), operative procedures except for left hepatectomy (*P* = 0.024), perioperative blood transfusion (*P* = 0.002), postoperative maximum T-bil >5.0 mg/dL (*P* = 0.002), T3/T4 stage (*P* = 0.014), positive lymph node (*P* = 0.035), microvascular invasion (*P* = 0.005), lymphovascular invasion (*P* = 0.043), and positive resection margin (*P* = 0.002). In multivariate analysis, perioperative blood transfusion (HR = 3.383 (95 % CI, 1.499–7.637), *P* = 0.002) and a positive resection margin (HR = 3.125 (95 % CI, 1.025–9.530), *P* = 0.045) were independent prognostic factors for OS. Kaplan–Meier survival curves gave median OS times after resection of 20.1 and 74.3 months with and without blood transfusion, respectively (Fig. 2).

**Table 4:** Clinicopathological features predicting overall survival

Variable	Number	5-year OS (%)	MST (month)	Univariate analysis	Multivariate analysis		
				*P* value	RR	95 % CI	*P* value
Gender							
Male	43	27.5	46.3	0.033*	1.519	0.524–4.399	0.441
Female	23	49.8	25.1				
Age							
≤70 years	42	47.4	56.4	0.280			
>70 years	24	16.5	29.8				
Preoperative jaundice							
No	19	60.5	NR	0.010*	1.936	0.607–6.177	0.264
Yes	47	24.5	26.0				
Preoperative serum CA19-9							
≤37 U/mL	32	54.7	74.3	0.004*	2.070	0.803–5.337	0.132
>37 U/mL	34	14.9	26.0				
Preoperative ICG-R15							
≤15 %	48	47.4	46.3	0.097			
>15 %	18	9.0	31.5				
FLR ratio							
>40 %	50	32.4	31.7	0.924			
≤40 %	16	44.4	53.5				
KICG of FLR							
>0.05	53	42.3	31.7	0.196			
≤0.05	13	20.0	24.3				
Preoperative PTPVE							
No	34	42.4	53.5	0.035*	0.962	0.376–2.463	0.936
Yes	32	28.8	23.1				
Liver resection							
Left	19	56.3	96.1	0.024*	2.332	0.628–8.668	0.206
Right^a^	47	25.1	28.2				
Operative time							
≤480 min	28	27.0	36.5	0.715			
>480 min	38	40.0	31.5				
Operative blood loss							
≤1500 mL	26	47.1	56.4	0.083			
>1500 mL	40	26.8	26.0				
Perioperative blood transfusion							
No	37	50.1	74.3	0.002*	3.383	1.499–7.637	0.003*
Yes	29	14.7	20.1				
Postoperative complication (Clavien–Dindo classification)							
Grades 0–2	35	42.5	53.5	0.065			
Grades 3–5	31	27.8	22.9				
Postoperative max. T-bil							
≤5.0 mg/dL	47	48.6	53.5	0.002*	1.155	0.484–2.757	0.746
>5.0 mg/dL	19	0.0	23.1				
Postoperative min. PT							
>60 %	46	32.4	31.7	0.762			
≤60 %	20	46.7	36.5				
Adjuvant chemotherapy							
Yes	30	24.3	26.0	0.914			
No	36	36.0	31.7				
T-stage (UICC)							
T1, T2	47	48.8	36.5	0.014*	0.954	0.369–2.466	0.922
T3, T4	19	0.0	24.3				
Lymph node status							
Negative	44	41.4	46.3	0.035*	0.826	0.286–2.383	0.723
Positive	22	23.8	22.9				
Tumor histological grade							
Papillary/well differentiated	16	27.6	31.5	0.684			
Moderate/poorly differentiated	50	37.3	33.5				
Microvascular invasion							
Negative	18	68.2	96.1	0.005*	2.016	0.525–7.736	0.307
Positive	48	14.5	29.8				
Lymphovascular invasion							
Negative	10	70.0	NR	0.043*	0.380	0.065–2.221	0.283
Positive	56	27.8	27.8				
Perineural invasion							
Negative	10	64.8	96.1	0.129			
Positive	56	29.1	31.5				
Margin status							
R0	54	42.3	36.5	0.002*	3.125	1.025–9.530	0.045*
R1	12	0.0	18.3				

*DFS* disease-free survival, *MST* median survival time, *RR* risk ratio, *95 % CI* 95 % confidence interval, *ICG-R15* indocyanine green retention rate at 15 min, *FLR* future liver remnant, *KICG* indocyanine green clearance rate, *PTPVE* percutaneous transhepatic portal vein embolization, *T-bil* total bilirubin, *PT* prothrombin time, *UICC* Union for International Cancer Control

*P < 0.05

^a^Including three right trisectionectomy and one central bisectionectomy

## Discussion

HCCA is a rare cancer with an estimated incidence of 0.8 and 1.2 per 100,000 in females and males, respectively. Recent developments in surgical techniques and perioperative management have led to a significant improvement in the resectability rate and survival for HCCA, but morbidity and a poor prognosis are still of concern. However, complete surgical removal of cancer tissues by surgical resection or liver transplantation is the only curative treatment, since there is no evidence of efficacy of chemotherapy or radiotherapy. In a recent review of reports of surgical resection for HCCA, Ito et al.^37^ found curative resection and 5-year survival rates of 14 to 95 % and 11 to 45 %, respectively, with morbidity of 14 to 76 % and mortality of 0 to 19 %. In our study, the curative resection rate using major hepatectomy with en bloc resection of the caudate lobe and extrahepatic bile duct was 81.8 %, which is relatively favorable compared with previous studies. Similar, we found a somewhat favorable 5-year survival rate of 35.7 %, morbidity of 47 %, and mortality of 1.5 % in the current series.

Many clinicopathological factors, including positive margin status, lymph node involvement, higher T-stage, moderate or poorly differentiated tumor grade, perineural invasion, microvascular invasion and lymphovascular invasion, can have a negative impact on long-term outcome after resection for HCCA.^6^,^7^,^16^,^17^,^19^,^38^^–^ 46 Of these factors, a positive margin status has frequently been identified as a risk factor associated with reduced survival in patients with HCCA. Complete resection with negative histologic margins is the only modifiable factor among potential risk factors, and thus is the primary goal of surgical treatment.^37^ Aggressive surgery with concomitant major hepatic resection is thought to be more effective for acquisition of a negative margin, compared with local resection.^6^,^7^,^42^,^46^,^47^ Consistent with this view, aggressive surgery in this study resulted in a relatively high R0 resection rate of 81.8 %, and cases with R0 resection had a significantly better prognosis compared with those with R1 resection.

The current study also indicated that a requirement for perioperative blood transfusion was a strong independent risk factor for both recurrence and poor survival after surgical resection for HCCA. In addition, intraoperative blood loss was significantly associated with blood transfusion requirements. Allogeneic blood transfusions are known to induce host immunosuppression. Some older reports have suggested some positive effects of blood transfusion, including a beneficial effect on graft prolongation after renal transplantation^48^ and reduction of recurrence of Crohn’s disease.^49^ In contrast, since Burrows et al. first reported that blood transfusion was associated with adverse oncologic outcomes after resection of colorectal cancer,^50^ many negative effects of blood transfusion have been described, including increased recurrence of colorectal cancer,^51^^–^ 57 gastric cancer,^58^^–^ 60 and pancreatic cancer.^61^^–^ 65 Therefore, avoidance of unnecessary blood transfusion is of particular clinical importance.

In liver surgery, there is a high incidence of blood transfusion because intraoperative blood loss is often large. Immunomodulation due to perioperative blood transfusion has been linked to cancer recurrence, particularly in surgery for hepatocellular carcinoma^66^^–^ 71 and colorectal liver metastases.^72^,^73^ However, developments in surgical techniques, perioperative management, and anesthetic protocols have markedly improved outcomes after liver surgery.^74^,^75^ Nevertheless, the procedure is often accompanied by substantial intraoperative blood loss and a requirement for blood transfusion perioperatively or in the postoperative course, especially in extended hepatectomy or surgery for liver cirrhosis.

Few studies have focused on the negative impact of blood transfusion on outcome after liver resection for HCCA. This type of hepatectomy is more complex and difficult to perform and more risky than that for hepatocellular carcinoma or liver metastasis because concomitant extrahepatic bile duct resection with lymph node dissection and biliary reconstruction are required for HCCA. This can lead to a large amount of blood loss and a need for blood transfusion in comparison with simple hepatectomy. Thus, the median blood loss in the current study was 1778.5 ml, which is a relatively large amount. A few studies have found that perioperative blood transfusion is a negative predictor for morbidity or mortality after resection for HCCA.^76^^–^ 78 The association between blood transfusion and long-term survival after resection for HCCA has only been examined in two previous studies,^79^,^80^ as far as we are aware. In 40 patients undergoing surgical resection for HCCA, Liu et al.^79^ found a significant association between blood transfusion and poor survival in univariate analysis, but blood transfusion could not be confirmed as an independent predictor in multivariate analysis. In a recent study of 83 patients with HCCA, Young et al.^80^ found that perioperative blood transfusion was a significant independent determinant of reduced survival after surgery by multivariate analysis. Similarly, in the present study, perioperative blood transfusion was a strong independent predictor for recurrence and poor survival after liver resection for HCCA. Thus, this study might be thought of as the second to show a significant independent correlation between blood transfusion and prognosis in patients with HCCA. One difference between this study and that of Young et al.^80^ is that all of our patients underwent major hepatectomy with caudate lobe and extrahepatic bile duct resection, whereas Young et al. included patients who underwent local resection alone, in addition to cases treated with aggressive surgery.^80^

The mechanism underlying the adverse effect of blood transfusion is unclear, but experimental and clinical studies have demonstrated that blood transfusion suppresses host immunity via reduction of natural killer cell activity and cytotoxic T cell function, increases suppressor T cell activity, and decrease helper/suppressor (T4/T8) lymphocyte ratios.^81^,^82^ Also, leaching of biologically active substance from cells into stored blood products occurs due to normal physiological aging and metabolic processes, and these leached bioactive substances have immunomodulatory effects that promote cell growth and angiogenesis, and may therefore have a direct effect on tumor growth.^83^ For this reason, the immunosuppressive effect of blood transfusion may play a major role in recurrence of a malignant tumor. Goeppert et al. recently showed that the presence of both intratumoral T and B lymphocytes is correlated with longer survival in cholangiocarcinoma, and that the prognosis was linked to inflammation. These data provide a solid basis for the understanding of the biological role of inflammatory infiltrates in cholangiocarcinoma and for functional and clinical studies exploring modulation of the inflammatory response in cholangiocarcinoma patients.^84^ The results indicate that an immunosuppressive status may affect recurrence and survival in patients with cholangiocarcinoma, even though the adverse effects of blood transfusion on host immunity are still unclear.

The current study has several limitations. First, it was performed retrospectively and not as a randomized controlled study, and included a relatively small number of patients who received blood transfusion. However, it is very difficult to design a prospective randomized trial to investigate the relationship between blood transfusion and HCCA outcome. Second, we did not analyze the association between transfusion quantity and survival because the small sample size of patients receiving blood transfusion may have resulted in a loss of statistical power. Third, we also did not analyze the influence of perioperative FFP transfusion on HCCA prognosis because almost all patients received administration of FFP during surgery or within at least 7 days postoperatively.

In summary, we identified perioperative blood transfusion and a histologic positive margin as strong independent predictors for a poor prognosis following aggressive surgery with concomitant major hepatectomy for HCCA. Thus, circumvention of perioperative blood transfusion is likely to play an important role in long-term survival for patients with HCCA. It is widely believed that an aggressive surgical approach to gain a negative margin is associated with a favorable prognosis. However, better long-term survival may be achieved through vigilant surgery to reduce blood loss and avoid blood transfusion when possible. More effective preoperative or postoperative adjuvant therapy and surgery for oncological control of advanced HCCA is also required to improve future outcomes.
